# A Retrospective Evaluation of Chemotherapy Overdoses in Dogs and Cats

**DOI:** 10.3389/fvets.2021.718967

**Published:** 2021-09-22

**Authors:** Margaret L. Musser, Kaitlin M. Curran, Brian K. Flesner, Chad M. Johannes

**Affiliations:** ^1^College of Veterinary Medicine, Iowa State University, Ames, IA, United States; ^2^Carlson College of Veterinary Medicine, Oregon State University, Corvallis, OR, United States; ^3^College of Veterinary Medicine, University of Missouri, Columbia, MO, United States

**Keywords:** antineoplastics, complications, miscalculation, treatment-associated deaths, adverse events, dosing errors

## Abstract

Chemotherapy overdoses (ODs) are severe complications that can occur following the use of antineoplastics. However, little is known about chemotherapy ODs in veterinary medicine. The goals of this retrospective study were to report the occurrence, type, and cause of known chemotherapy ODs in companion animal medicine. The American College of Veterinary Internal Medicine oncology and internal medicine listservs were solicited for chemotherapy OD cases in dogs and cats. An OD was defined as administration of a chemotherapy dose 10% higher than intended, or at a shorter interval than planned. Twelve non-anthracycline ODs in 11 dogs, and 3 cat ODs, were collected. Overdoses in dogs included carboplatin, cyclophosphamide, L-asparaginase, lomustine, mustargen, vincristine, and vinorelbine. The cat ODs included doxorubicin and vincristine. In dogs, the median OD was 2.1x (range: 1.2–10x) the intended dose. All dogs survived the OD and developed a variety of gastrointestinal and hematologic toxicities of varying grades. Both cats with a 2.4x vincristine OD died despite supportive care. The cat who received a 2x OD of doxorubicin survived the event, experiencing Veterinary Cooperative Oncology Group–common terminology criteria for adverse events (VCOG) grade I thrombocytopenia and anemia, and VCOG grade II neutropenia. Chemotherapy ODs appear to be rare in veterinary medicine and are typically 2–3xs the intended dose. Clinical effects include VCOG grade I and II gastrointestinal distress and VCOG grade III and IV hematologic effects. With appropriate supportive care, most patients will survive the event. Life-threatening events are more common in cats following vincristine ODs.

## Introduction

Optimal dosing of therapeutic agents aims to fall between the effective dose and maximum tolerated dose (1). This range often confers a margin of safety, and yet, clinically significant iatrogenic pharmaceutical overdoses (ODs) are common in human medicine (2). More problematic are chemotherapeutics, as a margin of safety is generally irrelevant: effective doses often cause significant adverse side effects, acceptable in human medicine when striving for a cure. Thus, even minor deviations from the intended dose can result in significant or life-threatening effects (3). The prevalence of chemotherapy errors in human medicine is not known as most published literature focuses on individual case reports and not encompassing cohort studies (4). Nevertheless, several agencies have attempted to decrease the probability of a chemotherapy OD by outlining standard practices to reduce the error rate (4, 5). Single agent chemotherapy ODs have been reported with lomustine (6, 7), oral and intrathecal methotrexate (8, 9), cisplatin (10), and various vinca alkaloids (11–13), among others. Overdose reports of multiple chemotherapeutic agents are even less common (14, 15).

Chemotherapy OD rates in veterinary medicine are similarly unknown and current literature is based on individual case reports. Dog chemotherapy ODs have been reported with cyclophosphamide (16, 17), 5-fluorouracil (18), lomustine (19), methotrexate (20, 21), vincristine (22) and doxorubicin (23). Cat chemotherapy ODs have been reported with vinca alkaloids (24, 25).

Given the lack of information regarding chemotherapy ODs and subsequent treatment recommendations in veterinary medicine, we sought to collect data from dogs and cats who experienced an OD with an ultimate goal of formulating recommendations for treatment following a chemotherapy OD. We also hoped this evaluation would provide information regarding the magnitude, clinical consequences, and types of ODs occurring in veterinary medicine to guide veterinary practitioners.

## Materials and Methods

The American College of Veterinary Internal Medicine (ACVIM) oncology and internal medicine listservs were solicited for chemotherapy OD cases. Participants collected case information electively by review of medical records and recorded cases via an electronic survey system (REDCap; Vanderbilt University, Nashville, TN). Clinicians who submitted cases may have searched their records using a variety of search terms, or simply recalled OD cases. Information collected included: signalment (species, breed, weight, age), tumor type being treated, comorbidities, chemotherapy administered, dosage (mg/m^2^), concomitant medications, why the error occurred, type of practice where the error occurred, adverse events observed and when they occurred, treatments administered and when they were initiated, and overall patient outcome. Adverse events were retrospectively reported by the submitting clinician according to the Veterinary Cooperative Oncology Group–common terminology criteria for adverse events v1.1 (26). A chemotherapy OD was based on a modified human definition and patients were included for analysis if dose administration was 10% over the intended dosage, or the chemotherapy was administered at a significantly shorter interval than was intended (5). The OD was reported as a ratio of administered drug to intended dosage (e.g., a dog that received 30 mg/m^2^ of vinorelbine instead of the intended 15 mg/m^2^ was classified as a 2x OD). If the intended dosage was unknown, the highest common published dosage was used to prevent overestimation of the OD (Table 1). An OD was defined to be discovered immediately if the error was identified within 24 h of chemotherapy administration. Prophylactic medications were those started after an OD was identified, but prior to the development of clinical signs. Descriptive statistical analysis was completed.

**Table 1:** Characteristics of dogs and cats experiencing chemotherapy overdoses.

**Breed**	**Diagnosis**	**Drug overdosed**	**Intended dosage**	**Administered dosage**	**Overdose**	**Typical maximum dosage used in veterinary medicine**	**Adverse events experienced (VCOG Grade)**	**Treatments administered**
Basenji	Thyroid carcinoma	Carboplatin	300 mg/m^2^	377 mg/m^2^	1.3x	300 mg/m^2^ (27)	Anorexia (III) Diarrhea (I) Lethargy (III) Nausea (III) Neutropenia (III) Vomiting (III)	Antibiotics not specified^†^ Dialysis Filgrastim^†^ Maropitant^†^ Metronidazole^†^
Bull mastiff mix	N/A	Cyclophosphamide	N/A (Cyclosporine was to be dispensed)	672	2.6x	250 mg/m^2^ (28)	Anorexia (II) Ascites (I) Hepatopathy (I-II) Lethargy (III) Polyarthritis (I)	Amoxicillin/clavulanic acid Esomeprazole Filgrastim IV Fluids Maropitant Metoclopramide S-Adenosylmethionine and silybin
Shih tzu	Grade II Soft tissue sarcoma	Metronomic cyclophosphamide	15 mg/m^2^/day	150 mg/m^2^/day	10x	25 mg/m^2^/day (29)	Anemia (IV) Anorexia (I) Diarrhea (II) Lethargy (IV) Nausea (I) Neutropenia (IV) Thrombocytopenia (IV) Vomiting (II)	Ampicillin sulbactam IV Fluids Enrofloxacin Maropitant Metronidazole Mirtazapine Ondansetron Pantoprazole Packed Red Blood Cells
Chihuahua mix	Multicentric lymphoma	Cyclophosphamide Vincristine	250 mg/m^2^ 0.5 mg/m^2^	300 mg/m^2^ 0.65 mg/m^2^^*^	1.2x 1.2x	250 mg/m^2^ (28) 0.75 mg/m^2^ (28)	Anemia (I) Cystitis (II) Neutropenia (III) None	Amoxicillin/clavulanic acid None
Cairn terrier	Intestinal large cell lymphoma	L-asparaginase	400 IU/kg	1,135 IU/kg	2.8x	400 IU/kg (30)	None	None
Shepherd mix	Histiocytic sarcoma	Lomustine	60 mg/m^2^	124 mg/m^2^	2.1x	90 mg/m^2^ (31)	Neutropenia (IV)	Activated charcoal with sorbitol^†^ Amoxicillin/clavulanic acid^†^ Apomorphine^†^ Maropitant^†^ Metronidazole^†^ Omeprazole^†^ S-Adenosylmethionine and silybin^†^
Not specified	Mast cell tumor	Lomustine	70 mg/m^2^	125 mg/m^2^	1.9x	90 mg/m^2^ (31)	Diarrhea (I) Neutropenia (IV) Vomiting (II)	Alpha lipoic acid Ampicillin sulbactam Enrofloxacin Filgrastim IV Fluids Maropitant Metronidazole Ondansetron Pantoprazole S-Adenosylmethionine and silybin
Bichon mix	Multicentric lymphoma	Mustargen	3 mg/m^2^	8.6 mg/m^2^	2.9x	3 mg/m^2^ (32)	Neutropenia (III)	Enrofloxacin^†^ Filgrastim^†^
Fox terrier	Immune-mediated thrombocytopenia	Vincristine	0.7 mg/m^2^	2.9 mg/m^2^	4.1x	0.75 mg/m^2^ (28)	Anemia (II) Anorexia (III) Diarrhea (III) Neutropenia (IV) Regurgitation (III) Tremors (II)	Ampicillin sulbactam Cisapride^†^ Cholestyramine^†^ Enrofloxacin Filgrastim^†^ Folinic acid^†^ Fresh whole blood Glutamic acid Metoclopramide^†^ Metronidazole Midazolam N-acetylcysteine^†^ Nasogastric tube and Vivonex nutritional support Ondansetron^†^ Pantoprazole^†^ Sucralfate
Samoyed	Multicentric lymphoma	Vincristine	0.7 mg/m^2^	1.13 mg/m^2^	1.6x	0.75 mg/m^2^ (28)	None	Ampicillin sulbactam Capromorelin Enrofloxacin^†^ Filgrastim^†^ Metoclopramide^†^ Ondansetron^†^ Sucralfate
Mixed breed	Pulmonary carcinoma	Vinorelbine	15 mg/m^2^	30.5 mg/m^2^	2x	15-18 mg/m^2^ (33)	Anorexia (III) Diarrhea (III) Ileus (III) Lethargy (II) Nausea (II) Neutropenia (IV) Vomiting (II)	Amoxicillin/clavulanic acid^†^ Ampicillin sulbactam Buprenorphine Enrofloxacin^†^ Filgrastim^†^ IV Fluids^†^ IV Lipid emulsion Maropitant^†^ Nasogastric tube Omeprazole Ondansetron^†^ Pantoprazole^†^
Domestic medium hair	Small and large cell gastrointestinal lymphoma	Vincristine	N/A (Vinblastine was prescribed but vincristine was prepared)	1.8 mg/m^2^	2.4x	0.5 mg/m^2^ (34)−0.75 mg/m^2^ (35)	Euthanized	Active warming IV Fluids Oxygen support
Domestic shorthair	Lymphoma (blood, pleural fluid)	Vincristine	N/A (Vinblastine was prescribed but vincristine was prepared)	1.8 mg/m^2^	2.4x	0.5 mg/m^2^ (34)−0.75 mg/m^2^ (35)	Euthanized	Activated charcoal^†^ Ampicillin sulbactam Dopamine Constant Rate Infusion Furosemide^†^ Maropitant^†^ N-acetylcysteine^†^ Ondansetron^†^
Domestic shorthair	Multicentric large cell lymphoma	Doxorubicin	1 mg/kg	2 mg/kg	2x	1 mg/kg (36)	Anemia (I) Neutropenia (II) Thrombocytopenia (I)	Cefovecin sodium^†^ Filgrastim Iron Dextran Maropitant

*IV, intravenous; VCOG, veterinary cooperative oncology group*.

* *Patient classified as an overdose as administered dose was 10% above intended dose for that patient*.

† *Treatments started prophylactically*.

## Results

Thirty-one OD incidents were collected from the ACVIM oncology and internal medicine listservs. Sixteen incidents described dog anthracycline ODs (mitoxantrone and doxorubicin) and will be reported elsewhere. Twelve non-anthracycline dog chemotherapy OD incidents (in 11 dogs) and three cat chemotherapy ODs were collected. No collected cases were excluded from analysis.

### Dog Chemotherapy ODs

The dog population consisted of 3 neutered males, 7 spayed females, and 1 intact male with an average weight of 17.2 kg (range: 2.34–42.8 kg). Patients were being treated for a variety of cancers (Table 1). Chemotherapy ODs were varied, with a median OD of 2.1x (range: 1.2–10x). Overdoses occurred because of a miscalculation of the chemotherapy dose (*n* = 2), an error in chemotherapy preparation (*n* = 2), incorrect weight (*n* = 3), chemotherapy was given to the wrong patient (*n* = 1), the owner gave the wrong dose of chemotherapy at home (*n* = 1), inadvertent administration of two oral doses of chemotherapy instead of one (*n* = 1), the pharmacist dispensed the wrong medication (cyclophosphamide instead of cyclosporine; *n* = 1), and the metered squared was converted based on pounds instead of kilograms (*n* = 1). The OD occurred in a specialty practice in 10 of the incidents, and at home in two. The OD was identified immediately in 7 of the 12 incidents. Clinical signs following the OD are outlined in Tables 1, 2.

**Table 2:** Adverse events and treatments in dogs following overdose of chemotherapy.

	**Grade**	**1–2x OD (*n* = 5)**	**2.1–3.9x OD (** ***n*** **= 5)**	**4–9.9x OD (** ***n*** **= 1)**	**10x (** ***n*** **= 1)**	**Common treatments administered**
Nausea	I					Maropitant
	II		1		1	Mirtazapine
	III	1				Ondansetron
	IV					Pantoprazole
	V					
Vomiting	I				1	Maropitant
	II	1	1			Ondansetron
	III	1				Pantoprazole
	IV					
	V					
Diarrhea	I	2				Metronidazole
	II				1	
	III		1	1		
	IV					
	V					
Anorexia	I				1	Capromorelin
	II		1			Cisapride
	III	1	1	1		Maropitant
	IV					Metoclopramide
	V					Mirtazapine
						Nasogastric tube and Vivonex nutritional support
						Ondansetron
						Pantoprazole
Lethargy	I					IV fluids
	II		1			
	III	1	1			
	IV				1	
	V					
Neutropenia	I					Ampicillin sulbactam
	II					Amoxicillin/clavulanic acid
	III	2	1			Enrofloxacin
	IV	1	2	1	1	Filgrastim
	V					
Thrombocytopenia	I					
	II					
	III					
	IV				1	
	V					
Anemia	I	1				Fresh whole blood
	II			1		Packed red blood cells
	III					
	IV				1	
	V					
Ileus	I					Buprenorphine
	II					
	III		1			
	IV					
	V					

In the 7 dogs where the OD was identified immediately, 6 started prophylactic medications including supportive gastrointestinal medications (*n* = 6), antibiotics (*n* = 5), and filgrastim (*n* = 5). Specific medications used for each patient are outlined in (Table 1). Additional treatments included IV lipid emulsion (8 mL/kg over 60 min; *n* = 1), dialysis (*n* = 1), and fresh whole blood (*n* = 1). In addition, in one patient who received an OD of lomustine, vomiting was induced with apomorphine (0.03 mg/kg once). This patient also received activated charcoal with sorbitol (20 mL/kg PO once) (Table 1). The remaining 5 dogs presented 2 days−2 weeks following the OD. These patients received supportive care directed at the clinical signs observed including maropitant (*n* = 1), metronidazole (*n* = 2), filgrastim (*n* = 1), antibiotics (*n* = 3), packed red blood cell transfusion (*n* =1), S-Adenosylmethionine and silybin (*n* = 1), alpha lipoic acid (*n* = 1), and esomeprazole (*n* = 1; Table 1).

Of the OD incidents where filgrastim was given prophylactically (*n* = 5), neutropenia still developed in four cases. This neutropenia was categorized as grade III or IV and lasted on average 7 days (range: 4–10; Table 1). One dog received filgrastim 2 days after the OD. This patient developed a grade IV neutropenia; the neutropenia lasted 7 days. A second dog received filgrastim following identification of an oral cyclophosphamide OD. Neutropenia did not develop in this patient. In the remaining five ODs, dogs did not receive filgrastim. Three of these incidents resulted in neutropenia [grades III and IV, lasting for an average of 9.6 days (range: 9–10)]. There was no numerical difference in grade of neutropenia or duration between those patients that received filgrastim and those that did not.

Of the OD incidents where gastrointestinal supportive medications were started prophylactically (*n* = 6; anti-nausea medications, and/or anti-diarrheal medications, and/or promotility agents, and/or proton pump inhibitors), 3 developed diarrhea [grades I and III lasting an average of 8 days (range: 7–9)] and anorexia [grade III lasting an average of 8.3 days (range: 6–10)], and 2 developed vomiting and/or nausea [grades II and III lasting an average of 8.5 days (range 7–10)]. In incidents where gastrointestinal medications were not started prophylactically (*n* = 5), 2 developed diarrhea [grades I and II lasting an average of 6 days (range: 4–8)], 2 developed anorexia [grades I and II lasting an average of 5.5 days (range: 5–6)], and 2 developed grade II nausea/vomiting lasting an average of 6.5 days (range: 6–7). In the final dog, the L-asparaginase OD was identified immediately but the patient did not receive prophylactic gastrointestinal supportive medications. It did not develop any adverse events, gastrointestinal or otherwise (Table 1).

One patient with immune-mediated thrombocytopenia received a 4x OD of vincristine. The OD was identified immediately, and supportive care was administered including IV fluids, metoclopramide, cisapride, ondansetron, pantoprazole, N-acetylcysteine, filgrastim, cholestyramine, and folinic acid. Three days following the OD the patient regurgitated bloody fluid and was acutely painful on abdominal palpation. An abdominal ultrasound was completed which revealed mild peritoneal effusion. Despite the prophylactic medications, this patient developed VCOG grade III diarrhea, grade III anorexia, grade III regurgitation, grade IV neutropenia, grade II anemia, and grade II tremors. Additional medications and treatments included whole blood transfusion, ampicillin sulbactam, enrofloxacin, sucralfate, glutamic acid, and Vivonex nutritional support. The patient was hospitalized for a total of 11 days and survived the OD. All clinical signs and hematologic abnormalities resolved by the time of discharge. The grade IV neutropenia was the most severe and longest lasting adverse effect, taking 9 days to resolve.

One patient received a 10x OD of metronomic cyclophosphamide (mCYC). Instead of receiving 4 mg of mCYC, 40 mg capsules were dispensed and given by the owner at home for 11 days. The patient was presented laterally recumbent, non-febrile, with pancytopenia (VCOG grade IV neutropenia, grade IV thrombocytopenia, and grade IV anemia). The patient also developed VCOG grade IV lethargy, grade II nausea, grade II diarrhea, grade I vomiting, and grade I anorexia 3–4 days after the OD. The patient was treated with IV fluids, ampicillin sulbactam, enrofloxacin, maropitant, ondansetron, pantoprazole, mirtazapine, metronidazole, and packed red blood cells. The patient was hospitalized for 12 days, but clinical signs did not resolve until day 14 following the OD. The patient survived and did not receive further chemotherapy.

### Cat Chemotherapy ODs

The 3 cats were either domestic short or medium haired. Two were neutered males, and one spayed female with an average weight of 3.4 kg (range: 2.6 to 3.9 kg). All had high grade lymphoma and were receiving an L-CHOP (L-asparaginase, cyclophosphamide, doxorubicin, vincristine, prednisolone) or CHOP protocol (Table 1). Two cats received at least a 2.4x OD of vincristine. Both cats were prescribed vinblastine, but vincristine was prepared instead. Both cats acutely decompensated within 36 h of chemotherapy administration. One cat arrived at an emergency clinic within hours of the OD hypothermic, hypotensive, and open mouth breathing. Supportive care with oxygen, fluids, and warming were administered, but the patient clinically decompensated. The second patient was prophylactically treated with maropitant (1 mg/kg IV q 24 h), ondansetron (1 mg/kg IV q 8 h), furosemide (0.5 mg/kg IV q 6 h), N-acetylcysteine (700 mg diluted to 5%, given IV over 15 min once), ampicillin sulbactam (30 mg/kg IV q 8 h), and activated charcoal (4 g PO 2 h post and 6 h post administration). However, the cat developed hypotension, bradycardia, and hypothermia 36 h after the OD. There was an acute decline in mentation despite continued supportive care with fluids and a dopamine constant rate infusion. Clinical and hematological adverse events were not evaluated in either patient as both owners elected humane euthanasia with the decline in clinical status.

The third cat received a 2x OD of doxorubicin due to a miscalculation. Cefovecin sodium (8 mg/kg SQ) was given immediately. This patient developed a grade II neutropenia, grade I thrombocytopenia, and grade I anemia. The patient was given supportive care including filgrastim (4.8 mcg/kg SQ on the day of the OD and the following day), iron dextran (20 mg/kg IM for 4 doses), and maropitant (2 mg/kg PO q 24 h). The cat was not hospitalized and survived the OD.

## Discussion

Chemotherapy ODs appear to be relatively uncommon in veterinary medicine but have the potential to cause serious morbidity or mortality. Within this patient population, ODs were generally 2–3 times the intended dosage and typically resulted in low grade to moderate gastrointestinal effects, and moderate to severe neutropenia, although individual variation did occur.

Within the group of patients reported here, 50% of the OD incidents involved vinca alkaloids (4 dog and 2 cat incidents). Individual case reports of vinca alkaloid ODs have been previously reported in the veterinary literature, most commonly involving cats (22, 24, 25). In the one dog case report, a 15-year-old mixed breed dog with multicentric lymphoma received a 10x OD of vincristine. The dog developed nausea, anorexia, hemorrhagic diarrhea, regurgitation, and megaesophagus, in addition to a moderate leukopenia and severe thrombocytopenia. Despite supportive care, the patient died of cardiopulmonary arrest 17 days post OD (22).

The dogs experiencing a vinca alkaloid OD in this study were administered 1.2–4x the intended dosage. Two of these patients had no adverse clinical signs develop, with only one receiving prophylactic supportive care (gastrointestinal medications, filgrastim, and antibiotics). The other two patients developed mild to moderate gastrointestinal effects and severe neutropenia, despite prophylactic and supportive care. Based on these cases, and the previous case report, following approximately 2–4x ODs of vinca alkaloids in dogs, mild to moderate gastrointestinal effects and severe neutropenia should be anticipated. Cats appear to have more severe reactions to moderate vinca alkaloid ODs. In this population, the two cats who received vincristine ODs experienced decompensation within 36 h of the OD and were humanely euthanized. This matches the previously published cat vincristine OD (25). Extreme care must be taken when dosing cat patients with vinblastine or vincristine, as a vincristine OD is likely to lead to death, even with a moderate OD.

Most ODs in this population resulted in mild to moderate gastrointestinal effects and severe neutropenia, with vincristine causing the most severe effects. However, given the variation in effects noted and drugs overdosed, general recommendations for care after an OD are difficult to define. Adverse effects did not differ significantly between those patients that received prophylactic gastrointestinal supportive medications and filgrastim and those that did not. However, no VCOG grade V events occurred in any dog, so perhaps this support prevented death following the OD. The study population was too diverse to strongly encourage or discourage the use of prophylactic medications, including filgrastim.

The patient who experienced a vinorelbine OD received IV lipid emulsion therapy within 8 h of the OD in an attempt to decrease the exposure of the patient to the OD. This technique has not been previously reported for chemotherapy ODs in humans or veterinary patients, but has been reported for other types of ODs (37). This therapy is often recommended for drugs that are highly lipophilic, such as vinorelbine, as it is thought IV lipid emulsions create a “lipid sink,” sequestering the lipophilic drug in a lipid partition within the blood, away from vital organs (37). Despite this treatment, the patient still experienced mild to moderate gastrointestinal signs and severe neutropenia but survived the OD. Additional studies are necessary to fully elucidate the impact of IV lipid emulsion therapy following chemotherapy ODs in veterinary medicine.

The data reported herein must be interpreted within the confines of a retrospective study. Although AEs were assigned VCOG grades when possible, these were interpreted from the clinical records and self-reported by the submitting clinicians. Thus, errors in grade assignment could have occurred, falsely increasing or decreasing the severity of the AE. The impact of prophylactic treatments, including filgrastim, are difficult to determine based on the limited data presented here. It is recommended that filgrastim be administered no sooner than 24 h after an OD to prevent severe neutropenia (38, 39). Information about the timing of filgrastim was also not collected and thus how, or if, filgrastim administration timing impacted the occurrence or severity of neutropenia in this population is unknown. As the intended dosage was not always known, it is also possible that the severity of the ODs reported may be higher if the intended dosage was below the highest common dosage used. The ODs reported here were solicited and self-reported, and therefore the true OD incidence within veterinary medicine is impossible to describe. Finally, a widely accepted definition of an OD does not exist in human or veterinary medicine. The definition used here was modified from one suggested in human medicine (5). Although a 10% OD may be a conservative definition and not result in serious clinical signs, an error of that magnitude in drugs with a narrow margin of safety should be recognized and implies the need for improved vigilance and processes to prevent more severe AEs from occurring.

Based on the results reported here, and the previous OD case reports, one should anticipate mild to moderate gastrointestinal effects and severe neutropenia following a chemotherapy OD, with the exception of L-asparaginase, in dogs. In cats, moderate vincristine ODs appear to be fatal, despite supportive care. If an OD occurs, it should be treated like other toxicities in veterinary medicine with the goals of providing supportive care, preventing further absorption, and administering specific antidotes when possible (40). Supportive medications to consider once an OD is identified include antinausea, antidiarrheal, and other gastrointestinal supportive medications. IV lipid emulsion therapy for lipophilic drugs, or therapeutic plasma exchange for highly protein bound chemotherapeutics, can be considered to decrease absorption of the drug (41, 42), although efficacy for these approaches has yet to be elucidated. Severe neutropenia is expected, and thus filgrastim administered no sooner than 24 h after the OD could be considered (38, 39), although impact on outcome is unknown. Most importantly, the causes of the ODs reported here were varied, implying that extreme vigilance (double and triple check systems) at all steps of chemotherapy dosing, preparation, and administration are needed to prevent ODs from occurring.

## Data Availability Statement

The raw data supporting the conclusions of this article will be made available by the authors, without undue reservation.

## Ethics Statement

Ethical review and approval was not required for the animal study because it was a retrospective study. Prior to treatment, informed client consent was obtained for each patient per the requirements of the participating institution. Written informed consent for participation was not obtained from the owners because this was a retrospective medical record review only.

## Author Contributions

MM and CJ prepared the digital survey for accurate data collection. Data was reviewed and descriptive statistics were prepared by MM, KC, BF, and CJ. Manuscript preparation was completed by MM and was reviewed by all authors.

## Conflict of Interest

The authors declare that the research was conducted in the absence of any commercial or financial relationships that could be construed as a potential conflict of interest.

## Publisher's Note

All claims expressed in this article are solely those of the authors and do not necessarily represent those of their affiliated organizations, or those of the publisher, the editors and the reviewers. Any product that may be evaluated in this article, or claim that may be made by its manufacturer, is not guaranteed or endorsed by the publisher.
